# A New Era of Diagnosis and Therapy in Acute Aortic Syndromes: The Mainz–Essen Experience (Part II)—Management and Outcomes

**DOI:** 10.1055/s-0041-1739466

**Published:** 2021-12-28

**Authors:** Eduardo Bossone, Riccardo Gorla, Brigida Ranieri, Valentina Russo, Heinz Jakob, Raimund Erbel

**Affiliations:** 1 Division of Cardiology, Antonio Cardarelli Hospital, Naples, Italy; 2 Department of Cardiology, IRCCS Policlinico San Donato, San Donato Milanese, Milan, Italy; 3 Cardiovascular Imaging Division, IRCCS SDN (Scientific Institute for Research, Hospitalization and Healthcare), Naples, Italy; 4 Department of Advanced Biomedical Sciences, Federico II University of Naples, Naples, Italy; 5 Department of Thoracic and Cardiovascular Surgery, West German Heart Center, University of Duisburg-Essen, University Hospital Essen, Essen, Germany; 6 Institute for Medical Informatics, Biometry and Epidemiology (IMIBE), University of Duisburg-Essen, University Hospital Essen, Essen, Germany

**Keywords:** acute aortic syndromes, Mainz–Essen, acute aortic dissection, hybrid operating room, heart team concept

## Abstract

Over the years, the cardiovascular department of Johannes Gutenberg University in Mainz-West-German Heart Centre in Essen (Germany) designed and implemented the hybrid operating room (2003) along with advanced endovascular and surgical procedures, including the frozen elephant trunk technique. For the study purpose, the Mainz–Essen experience on acute aortic syndromes was summarized by considering original articles from single-center or multicenter studies performed at West German Heart Centre, Essen, Germany, or at the cardiovascular department of Johannes Gutenberg University, Mainz, Germany. We present the 35-year-long Mainz–Essen research, education, and patient management journey in creating an integrated multidisciplinary “Aortic Center” in the heart of Europe.

## Introduction

Acute aortic syndromes (AAS) represent life-threatening medical emergencies needing a timely diagnosis and treatment ( Tables 1 and 2 ). 1 2 3 4 5 Current European guidelines recommend urgent surgical repair for Type-A AAS, medical therapy alone for uncomplicated Type B AAS, and thoracic endovascular aortic repair (TEVAR) for complicated Type B AAS. 1 Over the last decades, we have observed a substantial increase in surgical management for Type A acute aortic dissection (AAD; 79–90%) along with a significant decline of surgical in-hospital mortality (25–18%). On the other hand, no significant differences have been registered in Type B AAD overall in-hospital mortality (12–14%, *p* = 0.103). During this time, a larger number of complicated Type B AAD patients have been treated with endovascular interventions (7–31%; Supplementary Fig. S1 ). 3 4

**Table 1:** Acute aortic syndromes: definition and incidence 1 2

Acute aortic syndrome	Definition	Incidence a	Remarks
AD (85–95%)	Disruption of the medial layer provoked by intramural bleeding, resulting in separation of the aortic wall layers and subsequent formation of a TL and a FL with or without communication. In most cases, an intimal tear is the initiating condition	2.6–6	•The real incidence is difficult to define due to pre-hospital mortality and/or missing diagnosis •The incidence is higher in men and increases with age •Types: 67% Type A 33% Type B
IMH (10–25%)	Presence of hematoma in the media of the aortic wall in the absence of a FL and intimal tear	∼1.2	•Mainly in older patients •Types: 10–30% Type A 60–70% Type B •30–40% of Type A IMH evolved into AD
PAU (2–7%)	Ulceration of an aortic atherosclerotic plaque penetrating through the internal elastic lamina into the media	∼2.1	•Often multiple and different in size and depth. •More frequent in older age, male gender and in patients with atherosclerotic cardiovascular disease •Types: Rare in ascending aorta ∼17.5% aortic arch ∼68% descending aorta ∼14% thoracoabdominal transition

Abbreviations: AD, aortic dissection; FL, false lumen; IMH, intramural hematoma; PAU, penetrating aortic ulcer; TL, true lumen.

Note: Data from Erbel et al 1 and Bossone et al. 2

a All data are per 100,000 person-years.

**Table 2:** Classification systems of acute aortic syndromes 1

Classification		Description
Stanford	Type A	All dissections involving the ascending aorta irrespective of the site of tear
	Type B	All dissections that do not involve the ascending aorta; note that involvement of the aortic arch without involvement of the ascending aorta in the Stanford classification is labeled as Type B
DeBakey	Category I	Dissection tear in the ascending aorta propagating distally to include at least the aortic arch and typically the descending aorta
	Category II	Dissection tear only in the ascending aorta
	Category III	Dissection tear in the descending aorta propagating most often distally
	Category IIIa	Dissection tear only in the descending thoracic aorta
	Category IIIb	Tear extending below the diaphragm
Svensson	Class I	Classical dissection with true and false lumen
	Class II	Intramural hematoma or hemorrhage
	Class III	Subtle dissection without hematoma
	Class IV	Penetrating atherosclerotic aortic ulcer
	Class V	Iatrogenic or traumatic dissection
Time course: from symptom onset to presentation (Erbel et al 1 )	Acute	<14 days
	Subacute	15–90 days
	Chronic	>90 days

Note: Modified from Erbel et al. 1

We discuss the evolution, achievements, and the development that the Mainz–Essen center has experienced since the inception of a dedicated hybrid theater, as well as a dedicated team to treat the aortic pathologies ( Tables 3 6 7 8 9 10 11 12 13 14 15 16 17 18 19 20 21 22 23 24 25 26 and 4 5 27 28 29 30 31 32 33 34 35 36 37 38 39 40 41 42 ; Supplementary Table S1 and Supplementary Fig. S2 ).

**Table 3:** Studies addressing surgery in patients with Type A acute aortic dissection

Study (year)	Study type	Sample size ( *n* )	Age (y) Gender (M, F)	Topic	Mean follow-up	Main findings
Jakob et al (2008) 6	Retrospective single center	45 Type A AAD pts ( *n* = 23 conventional; *n* = 22 hybrid)	55.0 ± 15.0 18 M, 5 F ( *n* = 23 conventional) 57.0 ± 12.0 17 M, 5 F ( *n* = 22 hybrid)	Comparison of classical surgical treatment of Type A AAD vs. FET	48 ± 29 months (conventional) 23 ± 17 months (hybrid)	FET surgery is associated with higher rate of FL thrombosis at the distal edge of stent-graft compared with classical surgery
Tsagakis et al (2009) 16	Retrospective single center	41 pts (35 AD and 6 TAA)	60.0 ± 13.0 31 M, 10 F	Proximal endoleak with E-vita open hybrid stent-graft	17 ± 11 months	With FET proximal endoleak can be definitely avoided and in AD FL exclusion and shrinkage can be achieved
Tsagakis et al (2010) 7	Retrospective multicenter	68 AAD pts	58.0 ± 12.0 52 M, 16 F	FET in AAD	23 ± 17 months	3-year survival rate was 74%. FET promotes FL thrombosis around the stent-graft and below
Jakob et al (2010) 23	Retrospective multicenter	168 AD pts (29 AAD and 16 CAD)		Hybrid stent-grafting in Type I DeBakey AD	4 years	4-year survival was 72% in AAD vs. 94% in CAD. Freedom from reintervention was 90% in AAD and 75% in CAD
Tsagakis et al (2010) 24	Prospective single center	21 Type A AD pts (13 acute and 8 CAD pts)	60.0 ± 13.0 17 M, 4 F	Angioscopy in Type A AD	NA	Angioscopy is an important tool during surgery for decision making to apply open vision aortic cannulation, downstream stent grafting, landing zone control and need for ballooning
Tsagakis et al (2011) 17	Retrospective multicenter	106 acute and chronic AD	57.0 ± 13.0 82 M, 24 F	FET outcome with E-vita open hybrid stent-graft	20 ± 11 months	FET promotes FL thrombosis in the thoracic segment with acceptable perioperative risk
Pacini et al (2011) 8	Retrospective multicenter	240 FET pts (90 CAD pts)	57.0 ± 12.0 72 M, 18 F (CAD pts)	FET in chronic AD	20 ± 16 months	4-year survival and freedom from reintervention were 78% and 96%, respectively. Spinal cord injury seems unrelated to FL thrombosis
Tsagakis et al (2011) 25	Retrospective single center	118 AD pts	59.0 ± 13.0 76 M, 42 F	DeBakey's classification reflects late outcome and reintervention risk in AAD	24–33 months	5-year survival was 63% in Type I vs. 80% in Type 2. Freedom for reintervention was 100% in Type II, 82% in Type I with additional stenting and 53% in those without stenting
Benedik et al (2012) 20	Prospective single center	35 aortic walls	Age and gender NA	Dissectometer	NA/during surgery	Dissectometer is a useful tool to measure aortic wall consistence intraoperatively. A single sample of aorta is sufficient for assessment of aortic wall quality
Jakob et al (2012) 18	Prospective single center	77 acute and chronic AD pts	59.0 ± 11.0 57 M, 20 F	Long-term experience with E-vita open hybrid stent-graft	29 months	Complete thrombosis of thoracic FL was 92% for AAD, 91% for CAD, aneurysm exclusion was 100%. At 5 years, survival was 79%, freedom from major adverse events was 93%
Tsagakis et al (2013) 26	Prospective single center	124 Type A AAD pts	60.0 ± 13.0 80 M, 44 F	Hybrid OR concept for combined diagnostics, intervention and surgery in Type A AAD	NA	The hybrid OR concept enables exact diagnosis of coronary status and malperfusion sites and influences the choice of surgical and endovascular treatment without time delay
Tsagakis et al (2013) 19	Prospective single center	132 acute and chronic AD, and TAA	59.0 ± 11.0 95 M, 37 F	Overall Essen experience with E-vita open hybrid stent-graft	NA	5-year survival was 76% in AAD, 85% in CAD and 79% in TAA pts. Distal reintervention was infrequent and associated with low risk if indicated
Weiss et al (2015) 9	Retrospective multicenter	57 Type B AD pts	58.0 ± 12.0 42 M, 15 F	FET in complicated Type B AD	23 ± 19 months	FET is feasible for complicated Type B AD with involvement of aortic arch if TEVAR is contraindicated
Pilarczyk et al (2015) 21	Retrospective single center	105 aortic walls pts (51 patho and 54 normal pts)	67.0 ± 11.0 38 M, 13 F (patho) 67.0 ± 11.0 36 M, 18 F (normal)	Detection of aortic wall instability with dissectometer	NA	Dissectometer discriminates between stable and unstable aortic walls with good correlation to histological examination
Dohle et al (2016) 10	Retrospective single center	102 pts (70 AAD and 32 chronic AD)	60.0 ± 10.0 45 M, 25 F (AAD) 54.0 ± 13.0 29 M, 3 F (chronic AD)	Aortic remodeling after FET	47 ± 21 months	FET facilitates positive remodeling down to stent-graft level. Negative remodeling in ≥2 segments during follow-up is at risk for reintervention
Leontyev et al (2016) 11	Retrospective multicenter	509 AD and DA pts (350 AD pts)	64.1 ± 11.8 357 M, 152 F	Detection of predictors of in-hospital mortality after FET	NA	A distal landing zone other than T10 was an independent predictor of spinal cord injury. Hemodynamic instability, cerebral perfusion time >60 minutes, diabetes and peripheral vascular disease were predictors of in-hospital mortality
Dohle et al (2017) 12	Retrospective single center	63 Type A AAD pts	58.0 ± 10.0 43 M, 20 F	Impact of entries and exit on FL thrombosis and aortic remodeling	45 ± 26 months	Use of FET to treat Type A AAD facilitates positive remodeling at the stent-graft level and distally in two-thirds of patients
Iafrancesco et al (2017) 13	Retrospective multicenter	137 acute and chronic AD pts	59.0 109 M, 28 F	Aortic remodeling after FET	Median 32 months (IQR: 21–53 months)	FET promotes FL thrombosis and remodeling in descending thoracic aorta. False lumen status affects the diameter of aortic lumen. Chronic AAD shows higher rates of negative remodeling in descending thoracic aorta. Negative remodeling rate was similar between acute and chronic AAD in the abdominal aorta
Jakob et al (2017) 14	Retrospective single center	178 pts (96 AAD pts)	59.0 ± 11.0 125 M, 53 F	Long-term follow-up of E-vita open hybrid graft	36 ± 30 months	E-vita open hybrid stent-graft provides durable long-term performance. No interventions were necessary down to the end of stent-graft. Positive aortic remodeling at the stent-graft level was achieved in 92% of AAD, 82% CAD, full aneurysmal exclusion in 88%
Himpel et al (2017) 22	Retrospective single center	33 aortic roots and ascending aorta wall	65.0 ± 14.0 28 M, 5 F	Dissectometer	NA	Aortic root has a thin stable tissue, whereas ascending aorta wall was weaker despite its greater thickness
Tsagakis et al (2018) 15	Retrospective single center	286 pts (acute and chronic AD and TAA pts)	59.0 ± 11.0 199 M, 87 F	FET outcome in arch disease	NA	FET is the treatment of choice to achieve lasting results down to stent-graft end. FET combined with debranching enabling zone 2 arch repair improved the results

Abbreviations: AAD, acute aortic dissection; AD, aortic dissection; CAD, chronic aortic dissection; DA, degenerative or atherosclerotic aneurysm; F, female; FL, false lumen; FET, frozen elephant trunk; IQR, interquartile range; M, male; NA, not available; OR, operating room; patho, pathological; pts, patients; TAA, thoracic aortic aneurysm; TEVAR, thoracic endovascular aortic repair.

**Table 4:** Studies addressing thoracic endovascular aortic repair outcome in patients with Type B acute aortic syndromes

Study (year)	Study type	Sample size ( *n* )	Age (y) Gender (M, F)	Topic	Mean follow-up	Main findings
Herold et al (2002) 27	Retrospective single center	34 AAS and TAA pts	68.6 ± 7.0 27 M, 7 F	Outcome of TEVAR	8 months	TEVAR is safe and feasible, especially in emergencies
Eggebrecht et al (2003) 31	Prospective single center	10 PAU pts	71.3 ± 4.0 8 M, 2 F	Outcome in PAU	24.4 ± 10 months	TEVAR for PAU is safe and effective, also in case of aortic rupture
Eggebrecht et al (2004) 33	Retrospective single center	60 pts (3 AEF pts)	66.0 ± 10.0 46 M, 14 F	AEF	NA/5 weeks–10 months (death)	AEF is almost invariably fatal, treatment options are limited
Eggebrecht et al (2005) 36	Retrospective single center	17 pts (6 AAD pts)	65.2 ± 16.0 14 M, 3 F	Descending thoracic aortic rupture	Median: 23.7 months	TAA/AAD, mediastinal hematoma, >1 stent-graft, maximum aortic diameter >5 cm were important preprocedural denominators of death
Eggebrecht et al (2005) 28	Retrospective single center	38 Type B AAD pts	62.2 ± 10.8 32 M, 6 F	TEVAR outcome in Type B AAD	Median: 18 (range: 1–57) months	TEVAR is a safe alternative for pts with AAD. Preoperative clinical health status is the most important determinant of post-interventional outcome
Eggebrecht et al (2006) 32	Retrospective single center	22 PAU pts	69.1 ± 7.8 16 M, 6 F	Outcome in PAU	Median: 27 (range: 1–62 months)	Technical success was 96%. Acute and midterm mortality (2 years) was excellent, 61.9% at 5 years
Baumgart et al (2006) 29	Retrospective single center	84 AAS and TAA pts	64.0 ± 14.0 66 M, 18 F	Outcome of TEVAR	21 ± 18 months	Underlying aortic pathology and clinical health status affect outcome
Eggebrecht et al (2006) 37	Retrospective single center	97 AAS pts	64.4 ± 11.6 71 M, 26 F	Acute renal failure	NA/30 days–1 year/30 days–5 years	AKI has a significant adverse effect on 30-day and 1-year survival
Huptas et al (2009) 38	Prospective single center	27 Type B AAD pts ( *n* = 17 TEVAR pts; *n* = 10 medical pts)	60.0 ± 13.0 24 M, 3 F	Aortic remodeling after TEVAR	14 ± 6 months	TEVAR results in a significant increase in TL and decrease in FL volumes over time
Eggebrecht et al (2009) 34	Prospective single center	268 TEVAR pts (6 AEF pts)	63.5 3 M, 3 F	AEF	Median: 13.0 (IQR: 2.6–41.8) months	AEF is almost invariably fatal and should be suspected in case of new-onset fever and elevated inflammatory markers or hematemesis. Prompt diagnosis is crucial
Eggebrecht et al (2009) 39	Case report single center	4 SCI pts (1 pt case report)	3 M, 1 F (4 pts) 38 (1 pts)	Postoperative paraplegia	NA	Cerebrospinal fluid drainage may reverse completely delayed-onset paraplegia after TEVAR
Eggebrecht et al (2009) 40	Retrospective multicenter	63 TEVAR rAAD pts (48 pts all data)	Median age 56.5 (32–80) 31 M, 17 F	Retrograde ascending aortic dissection during or after TEVAR	During procedure up to 1,050 days	The incidence of rAAD is low (1.33%) with high mortality (42%); most rAAD occur after discharge and are associated with proximal bare spring stet-grafts. Surgery is the only option
Zahn et al (2013) 30	Prospective multicenter	191 AAS and TAA pts	64.5 ± 13.2 134 M, 57 F	Outcome of TEVAR	24.5 ± 27.7 months	Technical success (92%), endoleaks (8.5%). A high reintervention rate at 1 year was evident (7.2%)
Czerny et al (2014) 35	Prospective multicenter	36 pts AEF developed after TEVAR	Median age 69 (56–75) 27 M, 9 F	AEF	Days since initial TEVAR procedure, median: 90 (IQR: 30–150) days/1 year	Highest 1-year survival rate (46%) only with radical esophagectomy and aortic replacement
Kahlert et al (2014) 41	Descriptive single center	19 TEVAR pts	Median 59 (IQR: 19) 13 M, 6 F	Postoperative silent cerebral ischemia	Median: 5 (IQR: 3.5) days	TEVAR results in a high incidence of silent ischemic lesions (63%) on DW-MRI apparently without neurologic deficits
Jánosi et al (2015) 42	Retrospective single center	142 AAD pts	62.2 ± 12.6 96 M, 46 F	Thoracic aortic aneurysm expansion due to late distal stent-graft induced new entry	Mean 47.2 ± 37.1 months	dSINE is favored by a lower angle between distal stent-graft and aorta, by a higher taper ratio of the TL of the aorta and to a greater oversizing of the stent-graft in the distal landing zone
Jánosi et al (2016) 5	Retrospective single center	63 TEVAR pts	69.1 ± 11.5 40 M, 23 F	Outcome in PAU	45.6 ± 47.2 months	TEVAR is safe and effective. PAU patients often suffer from many comorbidities and atherosclerotic disease. PAU diameter >15 mm represents high risk for disease progression. D-d assessment may have an added value

Abbreviations: AAD, acute aortic dissection; AAS, acute aortic syndrome; AEF, aortoesophageal fistula; AKI, acute kidney injury; D-d, D-dimer; dSINE, distal stent-graft induced new entry; DW-MRI, diffusion-weighted magnetic resonance imaging; F, female; FL, false lumen; IQR, interquartile range; M, male; NA, not available; PAU, penetrating aortic ulcer; pts, patients; rAAD, retrograde ascending aortic dissection; SCI, spinal cord ischemia; TAA, thoracic aneurysm; TEVAR, thoracic endovascular aortic repair; TL, true lumen.

## Management and Outcomes of Acute Aortic Syndromes

Irrespective of subsequent therapeutic interventions (open surgery and/or endovascular), patients presenting with AAD should promptly receive initial aggressive medical therapy to decrease heart rate (<60 bpm), blood pressure (systolic blood pressure between 100 and 120 mm Hg), and dP/dT (the instantaneous rate of left ventricular pressure rise during early systole and is a surrogate marker of left ventricular contractility). In this regard, intravenous β-blockers (propranolol, metoprolol, labetalol, or esmolol) are considered first-line agents; nondihydropyridine calcium channel blockers (verapamil and diltiazem) are potential alternatives in truly β-blocker intolerant individuals. Sodium nitroprusside (if needed) should be given in conjunction with β-blocker to lower blood pressure. Intravenous opiate analgesia should be implemented to control pain and related activation of the sympathetic nervous system. 1

## Type A Acute Aortic Syndromes

Immediate open surgical repair is recommended for Type A AAD (mortality ∼22%). 1 3 4 The in-hospital mortality rate among Type A AAD patients managed medically is prohibitively high (∼55%). Reported reasons for not performing surgery usually consist of advanced age, significant multiple comorbidities, malperfusion, extensive stroke, patient refusal, and death prior to planned surgery. 1 3 4

### The Mainz–Essen Hybrid Operating Room Concept

During aortic emergencies, the ability to intervene expeditiously is essential for an optimal outcome. In the 1980s, few patients with suspected AAS were transferred with an external computed tomography (CT) scan to Mainz University Hospital. Thus, patients had to be diagnosed in house, but time loss due to patient transfer to and from a remote catheterization suite to the surgical department caused a more than 10% casualty toll due to rupture or malperfusion aggravation. This led to the concept of transferring the AAS suspected patient right away into the operative suite with immediate transesophageal echocardiography (TEE) as the only preoperative diagnostic measure on the operating table.

Over the last two decades, the hybrid operating room (OR) revolutionized the approach to vascular emergencies. An individualized patient management through modern imaging techniques and advanced therapeutic approaches can be performed, permitting a timely intervention in a single clinical room. This elaborate concept was proposed at the University Hospital Essen in 1995, accepted and integrated into the construction plans for building a new heart center in 1997, which finally was realized within the first heart center in a German University Campus in Essen, opening in 2003.

**Fig. 1:**
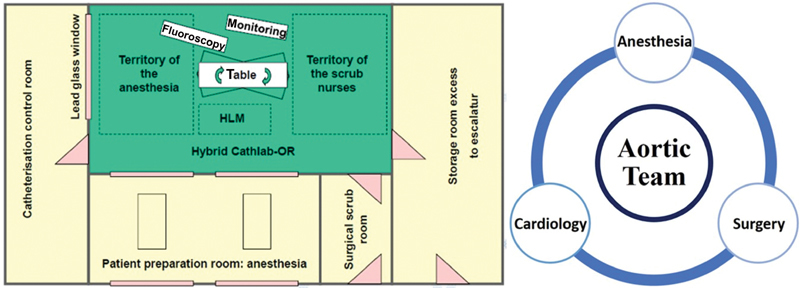
Essen hybrid room concept: invasive diagnostics and endovascular + open surgery. HLM, heart–lung machine; OR, operating room. 26

The whole catheterization area was constructed as an operating theater including laminar air flow, enabling absolute sterile conditions for complete diagnostic, and therapeutic options in cardiovascular emergencies. A 24/7 multidisciplinary “Aortic Team” (cardiac surgeons, cardiologists, and cardiac anesthesiologists) was initiated, which is the keystone feature to establish a timely diagnosis and to design the appropriate surgical procedure ( Fig. 1 ). 26

The catheterization table integrates all the elements of a surgical table, providing the possibility of performing surgery on the same table without moving the patient. 26 43

Nowadays, the European Society of Cardiology (ESC) guidelines consider the hybrid OR as the optimal environment for implementing new therapeutic options. 1 The Aortic Team, with the help of a multidisciplinary approach, modern imaging systems, careful planning, and professional expertise was able to develop new therapeutic approaches and modern surgical procedures. Jakob and Tsagakis 44 were the leading pioneers of the frozen elephant trunk (FET) technique, a modern surgery combining replacement of the ascending aorta, repair of the aortic arch, and endografting of the proximal descending aorta 7 15 44 ( Supplementary Fig. S3 6 ; available in the online version).

**Fig. 2:**
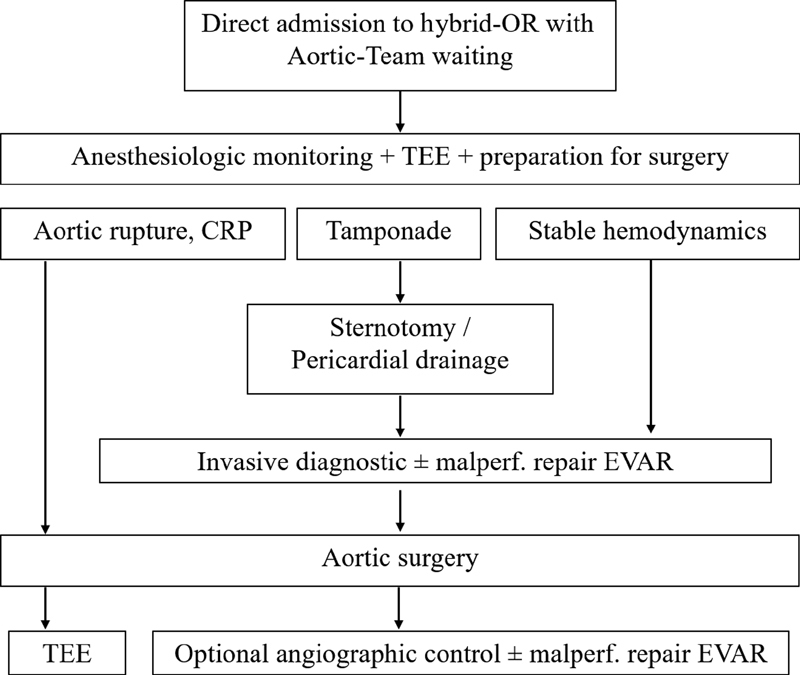
Essen algorithm for hybrid operating room concept for acute Type A aortic dissection management. CRP, C-reactive protein; EVAR, endovascular aneurysm repair; OR, operating room; TEE, transesophageal echocardiography. Image courtesy: Tsagakis et al. 26

It could be demonstrated that in-hospital mortality did not become worse by this more complex surgical approach; rather long-term outcomes improved in terms of fewer reinterventions/reoperations downstream. 9 14 23 Moreover, proof could be generated that positive remodeling and false lumen thrombosis take place in more than 95% of acute, and in more than 90% of chronic Type I aortic dissection cases, in the region around the descending aorta stent-graft. 10 12 The international E-vita registry, inaugurated by the Essen group, with the participation of 20 European cardiothoracic centers, confirmed the results from Essen. Indication for the use of FET was able to be extended, leading, based on observed results, to modifications in surgical strategies throughout all participating centers. 7 8 9 11 17 45 46 Based on these prerequisites, additional aspects of surgical treatment options and safety tools for implantation of FET could be elaborated in Essen, including the very fast and safe cannulation technique in threatening emergency situations, and the nowadays angioscopic evaluation of the thoracoabdominal aorta during hypothermic circulatory arrest, to control the FET landing zone deep down in the surgically invisible mid-descending thoracic aorta. 24 47 With this advanced knowledge about the pathophysiology and pathomorphology of acute Type I or II aortic dissection, it became possible to differentiate the indication for FET or lack of indication. When the dissection process stops within the ascending aorta (the classic DeBakey Type II), or within the arch (the Essen modified Type II classification), hemiarch and total arch replacement is sufficient without FET, resulting in stable long-term results without the need for late reintervention. 25 Thus, for patients with AAS, a significant improvement in the management of acute aortic emergencies was achieved, including aortic dissection, leading to the so-called “Essen hybrid” algorithm ( Fig. 2 ). 26

All patients with suspected or proven aortic dissection are transported directly into the hybrid OR. Furthermore, with the hybrid OR, it is possible to investigate a potential malperfusion site without time loss. The coronary, cerebral, visceral, and peripheral vascular branches are investigated through angiography in stable patients, while the anesthesiologist is instrumenting the patient and controlling blood pressure, the scrub nurse is preparing the instrument tables, and the surgeon is standing by to intervene immediately in case of a complication.

**Fig. 3:**
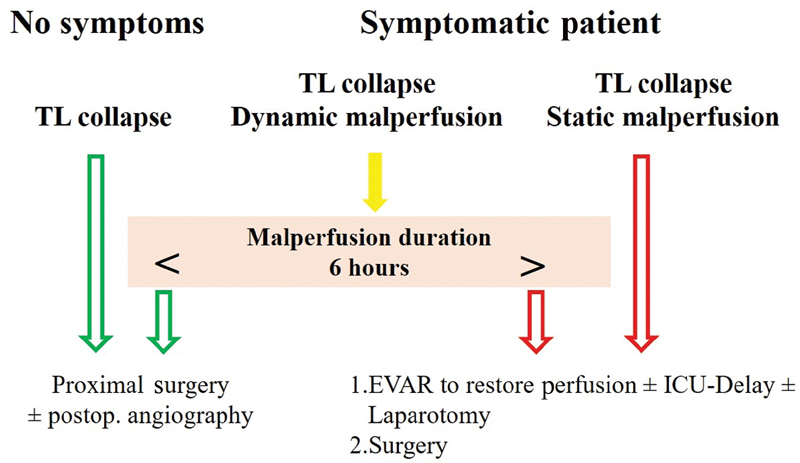
Essen algorithm for visceral malperfusion. Treatment planning in the hybrid operating room. EVAR, endovascular aneurysm repair; ICU, intensive care unit; TL, true limen. 26

An elaborate malperfusion regimen has been developed over the past 10 years enabling decision-making, for example, whether or not to treat severe visceral malperfusion prior to surgery or afterward ( Fig. 3 ). 26 Thus, it becomes possible to restore end-organ perfusion prior to surgery by endovascular means. In case of prolonged (>6 hours) total or subtotal malperfusion with elevation of liver enzymes and lactate and/or in symptomatic patients, an intensive care unit controlled delay to definitive surgical treatment of the underlying thoracic aortic pathology can be chosen in hemodynamically stable patients under continuous TEE control. 48 49

The Essen hybrid OR concept demonstrates the possibility of individualized management for Type A AAD based on the Penn classification, because advanced diagnostics and therapeutics can be performed without delay in a single clinical venue. 50 51 The hybrid OR concept and associated developments resulting from its improved capabilities for diagnostics, intervention, and differentiated surgical options enables individualized, patient-oriented optimal treatment options. The authors believe that the associated clinical outcome is better compared with the conventional approach, though not yet reaching statistical significance. 26 51

## Management of Type B Acute Aortic Syndromes

Definitive management of uncomplicated Type B AAD consists of close surveillance (clinical and imaging) and aggressive medical therapy (in-hospital mortality ∼11%). 1 3 4 Nowadays, according to the ESC 1 and more recent European Society for Vascular Surgery (ESVS) 52 guidelines, TEVAR is recommended as the therapy of choice in complicated Type B AAD, 1 defined by persistent or recurrent pain, uncontrolled hypertension despite full medication, early aortic expansion, malperfusion, and signs of rupture (hemothorax, increasing periaortic, and mediastinal hematoma). 1 Optimal positioning of stent-grafts leads to thrombus formation in the false lumen and initiates aortic healing with complete restoration of normal anatomy in many cases, in case of continuing patency of the false lumen, slow but continuous aortic enlargement can result. 38 Implantation of additional bare metal stents was introduced as “PETTICOAT” stenting by Nienaber et al. 53 In parallel, the later called “STABILISE” technique has been introduced by Hofferberth et al 54 in the first decade of this century, using a balloon fracturing method within the distal portion of the covered descending stent-graft to rupture the separating lamella, protected by noncovered stents down to aortic bifurcation. Thus, perfect relamination of the stented aorta could be achieved. 54 It should also be highlighted that TEVAR may induce a positive aortic remodeling at the level of the thoracic aorta, with continuous gain in true lumen volume over time, as well as progressive reduction of false lumen volume, in contrast to medically treated uncomplicated Type B AAD patients. This finding has relevant clinical implications implying potential therapeutic benefit of TEVAR over medical therapy alone in uncomplicated Type B dissection which warrants further randomized studies. 55 56 Thus, all patients with suspected or proven Type B AAD are treated in the hybrid room initially, and in ensuing diagnostic and therapeutic sessions, as well. In case of persisting true lumen compression, noncovered stents are placed downstream in addition to the covered stent at the descending aortic position. Finally, open surgery may be considered a treatment option in the case of complicated Type B AAS, not suitable to TEVAR (in-hospital mortality rate ∼30%). 1 3 4

### Studies Addressing Thoracic Endovascular Aortic Repair Outcome in Patients with Type B Acute Aortic Syndromes

Already, the first case of Type B AAD which was detected by TEE opened the question of further therapy. Most striking was the imaging of the entry tear at the descending aorta just distally to the origin of the subclavian artery which had not been reported previously. 57 Closure of the tear seemed to be the logical consequence of this finding, but at that time, few surgeons were available who were experienced in aortic surgery. At that time, paraplegia after surgery was observed quite often. In this regard, feasibility and safety of TEVAR was assessed by Eggebrecht et al 28 in 38 aortic dissection patients, with excellent overall in-hospital survival (89%), 30-day mortality (97.4%), 1-year (80.4%), 2-year (73.2%), and 4-year survival (54.9%). Intimal entry could be sealed in all cases and, notably, a preoperative poor clinical health status was the most important determinant of postinterventional mortality. Excellent technical success rates (>90%) were confirmed also by other single-center and multicenter reports. 27 29 30

Zahn et al 30 enrolled 191 patients undergoing TEVAR including 104 Type B AAD, 91 thoracic aortic aneurysm, 20 Type B acute intramural hematoma, 16 Type B acute penetrating aortic ulcer (PAU), and 6 traumatic aortic ruptures. A high technical success rates (92.1%) along with acceptable in-hospital complication rates were registered (14.1%; 15 patients presented endoleaks [Types I–III], 7 patients stroke, and 3 paraplegia; only 6 patients needed a reintervention). The in-hospital mortality was 5.5%. The 1-year reintervention and mortality rates were 7.2 and 11.4%, respectively ( Supplementary Fig. S4 ).

Jánosi et al 5 analyzed 63 patients with complicated PAU (42 symptomatic and 22 with rupture) who underwent TEVAR ( Supplementary Fig. S5A ). Among these patients, 33 received TEVAR within 14 days of diagnosis, while the other 30 were treated with an average interval between diagnosis and intervention of 40 ± 39 days. An excellent technical success rate (98.4%) along with an in-hospital mortality of 7.9% was observed. Long-term overall survivals estimated at 1, 5, and 10 years were 88.4, 82.2, and 65.7%, respectively. During follow-up, 19.0% of the patients needed a secondary intervention due to new complications or late endoleaks. Furthermore, there was no significant difference in long-term survival between patients who had undergone TEVAR for PAU and the historical cohort of patients treated with TEVAR for classic false lumen Type B AAD ( Supplementary Fig. S5B ). 5 Interestingly, a PAU diameter of >15 mm and higher D-dimer levels appear to play a role in the identification of patients at risk and with disease progression. 5

### Thoracic Endovascular Aortic Repair–Related Complications

Several important complications related to the procedure may affect the postoperative course of TEVAR patients and should therefore be promptly suspected and adequately managed by clinicians.

- Acute kidney injury: postprocedural acute kidney injury (AKI) is a complication related to catheter-based interventional procedures that use intra-arterial contrast agents. The prevalence of AKI, defined as an increase ≥25% and/or 0.5 mg/dL in preprocedural serum creatinine at 48 hours after TEVAR, has been reported in 38.9% 58 and 34% 37 of patients with AAS. Development of AKI was closely related to preoperative hemoglobin levels and postoperative hemoglobin drop, and these factors were independent predictors of in-hospital mortality. Additionally, malperfusion at admission, higher loads of contrast medium, and diabetes mellitus were important risk factors for AKI. Therefore, the use of an isoosmolar contrast medium, as well as measures aiming at preventing procedure-related bleeding and access site complications and careful monitoring of fluid status to avoid excessive hemodilution should be pursued. The prognostic impact of AKI on follow-up mortality remains to be clarified.
- Postimplantation syndrome (PIS): TEVAR induces elevations of inflammatory and coagulative biomarkers (white blood cells, C-reactive protein, fibrinogen, and D-dimer). 59 In ∼16% of patients, the inflammatory response leads to clinical manifestations, including fever with negative blood cultures sometimes accompanied by back or thoracic pain within 48 to 72 hours after TEVAR. This is known as PIS. 60 Diagnostic criteria of PIS include at least two of the four systemic inflammatory response syndrome criteria (fever >38°C, white blood cell count >12,000/μL). An increase in C-reactive protein >10 mg/dL may be an additional criterion with important prognostic implications in addition to interleukin-6 which seems to be PIS specific ( Supplementary Fig. S6 ). 61

Interestingly, although PIS did not affect in-hospital outcome, it was associated with higher rates of major adverse events (aortic rupture, endoleak Type 1, and reintervention), but not of mortality at 4-year follow-up. 61 However, the role of corticosteroids as therapeutic agents aiming at preventing complications during follow-up should be validated in future studies.

- Aortoesophageal fistula: aortoesophageal fistula (AEF) is a rare and dreaded complication following TEVAR which requires a high index of suspicion for a prompt diagnosis. The reported prevalence is 1.9% and it is characterized by new-onset fever with elevated inflammatory biomarkers (81%), hematemesis (53%), and shock (22%). 33 34 Prompt diagnosis with CT (new mediastinal mass) or esophagogastroduodenoscopy (visualization of the stent-graft) is of paramount importance ( Supplementary Fig. S7 ). 34 If medically treated, this condition is invariably fatal, whereas combined radical esophagectomy and aortic replacement has the highest 1-year survival (43%). 35
- Symptomatic spinal cord ischemia: its prevalence is up to 5% and it occurs more frequently in emergency scenarios. Prolonged intraoperative hypotension combined with simultaneous closure of at least two of four independent spinal cord supplying territories (subclavian, intercostal, lumbar, and hypogastric) had the strongest association with symptomatic spinal cord ischemia. This finding emphasizes the importance of preserving the left subclavian artery during TEVAR to maintain posterior cerebellar perfusion. In contrast, extensive coverage of intercostal arteries by a thoracic stent-graft alone seems not relevant. 62 The onset of symptomatic spinal cord ischemia may occur up to several hours after TEVAR procedures in up to 50% of cases. In addition to augmentation of mean arterial pressure, cerebrospinal fluid drainage was effective in complete reversal of patient's symptoms but can lead to serious complications such as spinal headache and epidural/subdural hematoma.
- Silent cerebral ischemia: the risk of clinically apparent periprocedural stroke after TEVAR has been reported between 2 and 6% and is related to intravascular manipulation of guidewires, catheters, and delivery systems, with subsequent dislodgement of atherosclerotic plaque debris. However, new silent cerebral lesions have been identified in 63% of patients in a diffusion-weighted magnetic resonance imaging (MRI) study, with no differences according to the indication for TEVAR, overstenting of the left subclavian artery (provided the exclusion of patients with left dominant vertebral artery), stent landing zone, and previous aortic surgery. Further studies are needed to identify patient- and procedure-related risk factors for cerebral embolism during TEVAR. 41
- Retrograde ascending aortic dissection: although the prevalence of this potentially lethal complication is very low (1.33%), retrograde ascending aortic dissection (RAAD) is associated with high mortality (42%). It can occur preoperatively (21%), during TEVAR (15%), or more frequently during follow-up (65%). In the majority of cases, RAAD is related to the use of stent-grafts with bare proximal springs. Direct evidence of induced stent-graft has been confirmed injury at surgery or necropsy. Less frequently RAAD may be attributed to disease progression or manipulation of guidewires and sheaths during the procedure ( Supplementary Fig. S8 ). 40 Presentation symptoms include chest pain, syncope, or stroke, but patients can be asymptomatic in 25% of cases. Surgical repair is the only option if dissection is related to stent-graft injury, whereas a conservative treatment may be feasible if dissection is wire related. 40
- Distal stent-graft induced new entry: the reported prevalence is 6.3%; this can occur up to 6 years after the index procedure, and patients are often asymptomatic. Once the diagnosis is made on CT angiography, close follow-up at every 3 months should be recommended to evaluate the progression and formation of the distal aneurysm caused by expansion of the false lumen. Indications for reintervention include persistent enlargement of the false lumen, symptomatic state, and signs of rupture. Patients with greater oversizing of the stent-graft at the distal landing zone, as well as those with a more acute angle between the bottom edge of the stent-graft and aorta were at higher risk of stent-graft induced new entry. Therefore, especially in older dissection, distal oversizing should be kept to a minimum or avoided, or tapered stent-grafts may be preferred. Technical success of reintervention was 100%. 42

## Long-Term Follow-up Clinic

AAS is a life-long medical entity involving the entire aorta and its branches (holistic view) implying a substantial risk for redissection, aneurysm formation, and rupture. 1 Thus, all patients affected by AAS should undergo close clinical and imaging follow-up (before discharge and at 1, 3, 6, and 12 months, and annually thereafter [biannually in the case of very stable patients]). Although CT is the most used imaging technique, MRI should be preferred (especially in young patients; being ionizing radiation free). In Essen, a weekly outpatient clinic is held and new patients are screened, as well as all patients having undergone aortic surgery are seen annually or biannually thereafter, including imaging. Thus, exact knowledge of the patients' status and their aorta is available to determine whether or not additional therapeutic steps are necessary. 63 Optimal blood pressure and heart rate control (blood pressure <120/80 mm Hg, heart rate <60 bpm) represent a cornerstone of medical treatment (first line: long-acting β-blockers and second line: angiotensin-converting enzyme inhibitors or angiotensin receptor blockers). Secondary prevention measures tailored on an individual patient basis should be implemented to reduce the burden of cardiovascular risk factors. 1 To ensure a perfect follow-up, the Aortic Center in Essen organized regular patient visits; patients are seen by members of the cardiology and cardiovascular surgery team, including comprehensive CT or MRI follow-up imaging but also addressing blood pressure control and medical events.

## Conclusion

The integrated multidisciplinary endovascular and surgical approach to AAS, along with the design and implementation of the hybrid OR concept, have represented a “Rosetta Stone” of AAS therapeutic interventions, paving the way for investigators to follow. In this regard, endovascular treatment of clinically stable Type B AAS remains an open question to be answered by future studies. Serologic and imaging biomarkers may play a key role on early detection of unstable disease in otherwise clinically stable patients.
