# Supplementary material for: A New Era of Diagnosis and Therapy in Acute Aortic Syndromes: The Mainz–Essen Experience (Part II)—Management and Outcomes
Source: Aorta (Stamford). 2021 Dec 28;9(6):201–14. doi: 10.1055/s-0041-1739466 (PMC8714317; doi:10.1055/s-0041-1739466)
Supplement: Supplementary file 1 — Supplementary Material [file 10-1055-s-0041-1739466-s200032.pdf]

A

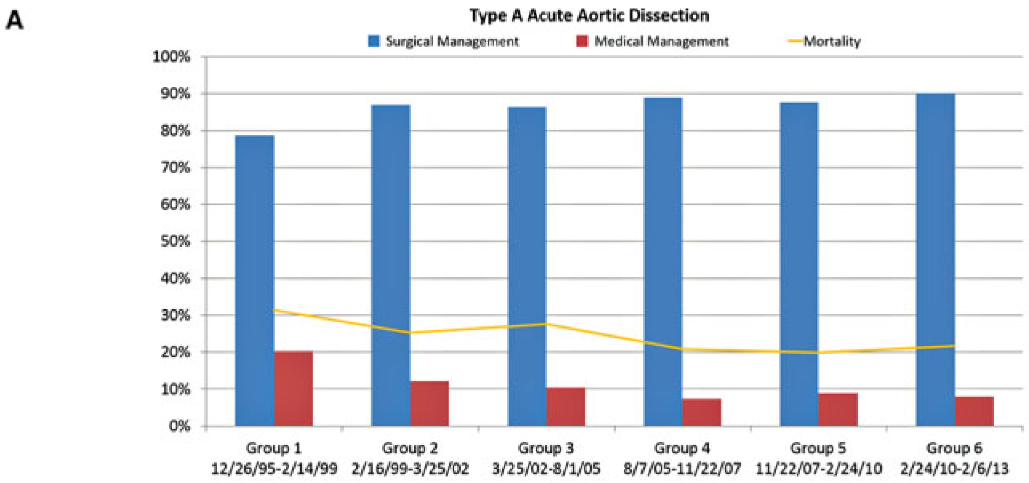

							p-value	Trend p-value
Overall Mortality	154 (31.4%)	119 (25.3%)	140 (27.6%)	99 (20.8%)	103 (19.8%)	106 (21.7%)	<0.001	<0.001
Surgical Mortality	96 (25.0%)	80 (19.6%)	97 (22.1%)	76 (17.9%)	72 (15.8%)	81 (18.4%)	0.015	0.003
Medical Mortality	54 (54.5%)	37 (64.9%)	34 (64.2%)	17 (48.6%)	24 (52.2%)	22 (56.4%)	0.512	0.626

B

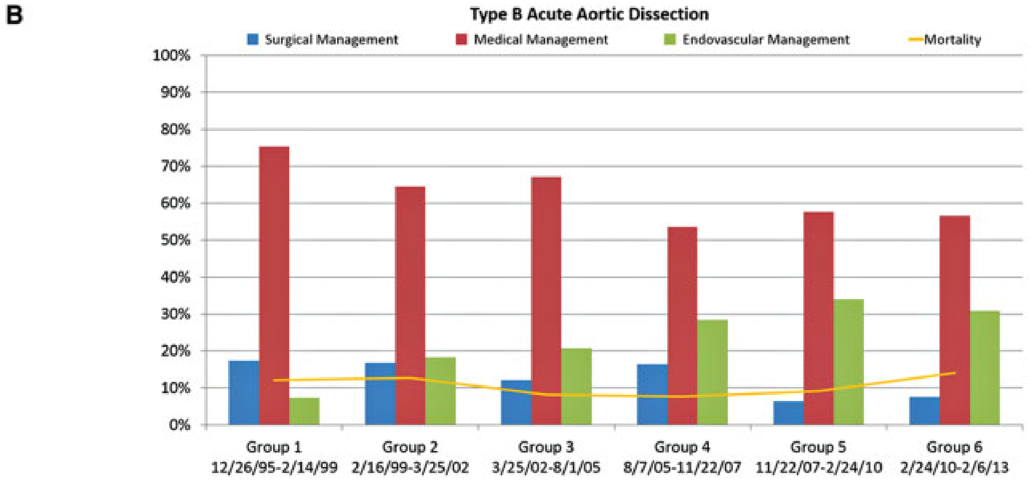

							p-value	Trend p-value
Overall Mortality	30 (12.1%)	34 (12.7%)	19 (8.2%)	20 (7.7%)	20 (9.1%)	35 (14.1%)	0.103	0.915
Surgical Mortality	13 (30.2%)	12 (26.7%)	1 (3.6%)	3 (7.0%)	0 (0.0%)	4 (21.1%)	0.003	0.007
Medical Mortality	17 (9.1%)	16 (9.2%)	11 (7.1%)	10 (7.1%)	9 (7.1%)	17 (12.1%)	0.630	0.705
Endovascular Mortality	0 (0.0%)	6 (12.2%)	7 (14.6%)	7 (9.5%)	10 (13.3%)	12 (15.6%)	0.528	0.216

Supplementary Fig. S1 Aortic dissection: management and mortality over time in the International Registry of Acute Aortic Dissection registry. Image courtesy:Evangelista et al.[3]

 © 2021. The Author(s).

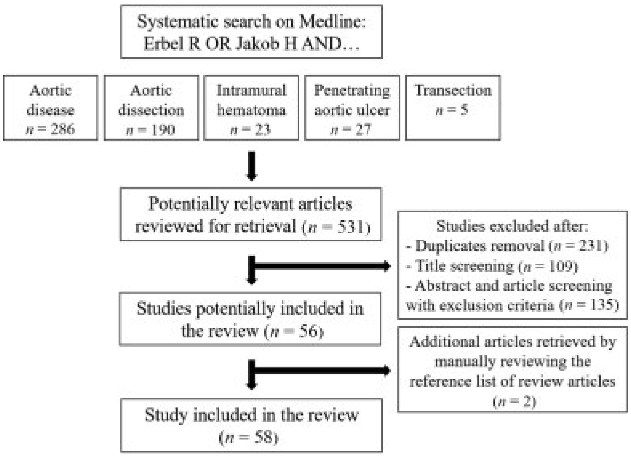

Supplementary Fig. S2 Study flowchart.

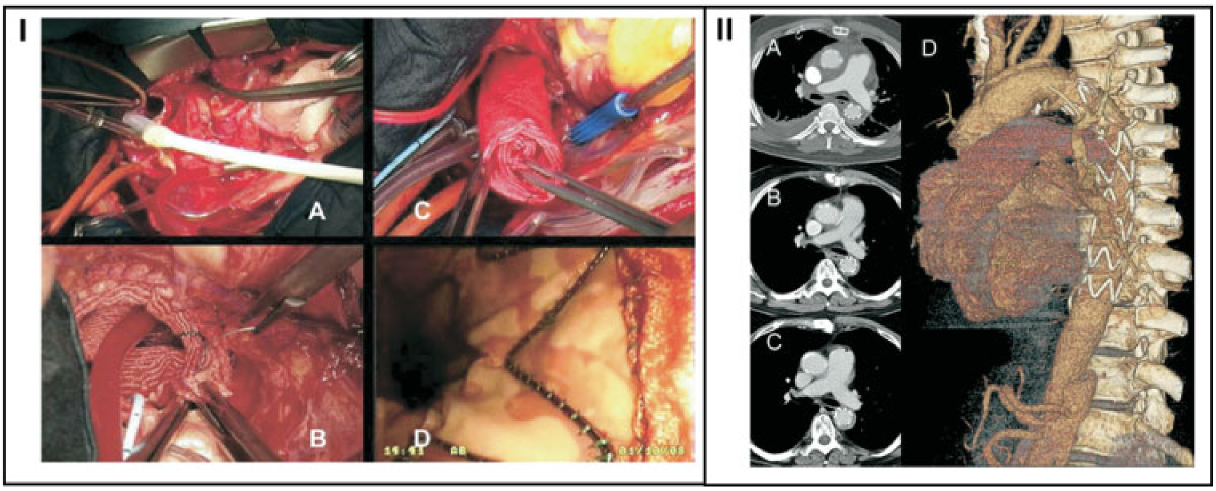

Supplementary Fig. S3 The hybrid prosthesis into the aorta. (Part I): (**A**) Insertion of the hybrid prosthesis into the descending aorta. (**B**) Distal anastomosis between the integrated polyester prosthesis and descending aorta. (**C**) Unfolding of the attached polyester graft for arch island anastomosis. (**D**) Angioscopic view through the arch prosthesis demonstrating direct continuity between the arch prosthesis and the descending stent graft. (Part II): A 52-year-old man after E-vita open stent grafting for acute Type A aortic dissection with durable thrombosis/shrinking of the false lumen in the descending aorta. Computed tomography scans (**A**) 10 days postoperatively, (**B, D**) after 6 months, and (**C**) after 12 months. Image courtesy:Jakob et al.[6]

 © 2021. The Author(s).

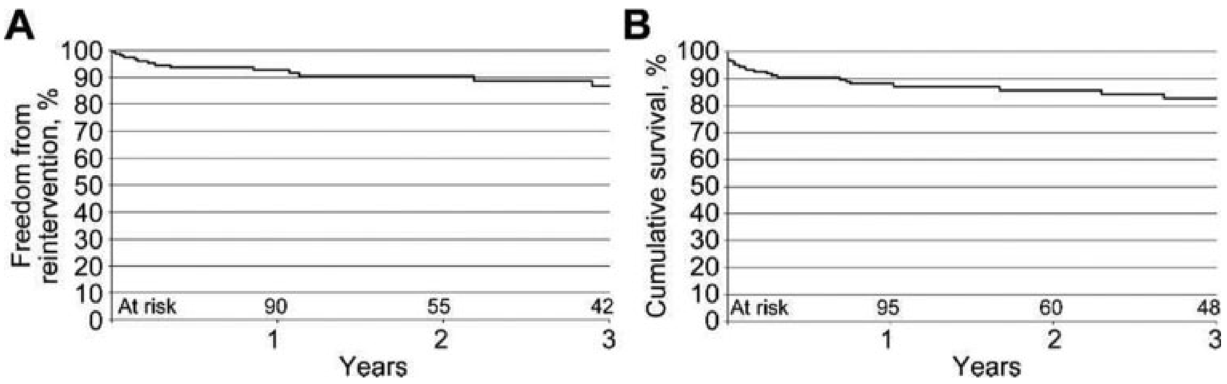

Supplementary Fig. S4 Kaplan–Meier curves for (**A**) freedom from aortic reinterventions and (**B**) survival. Image courtesy:Zahn et al.[30]

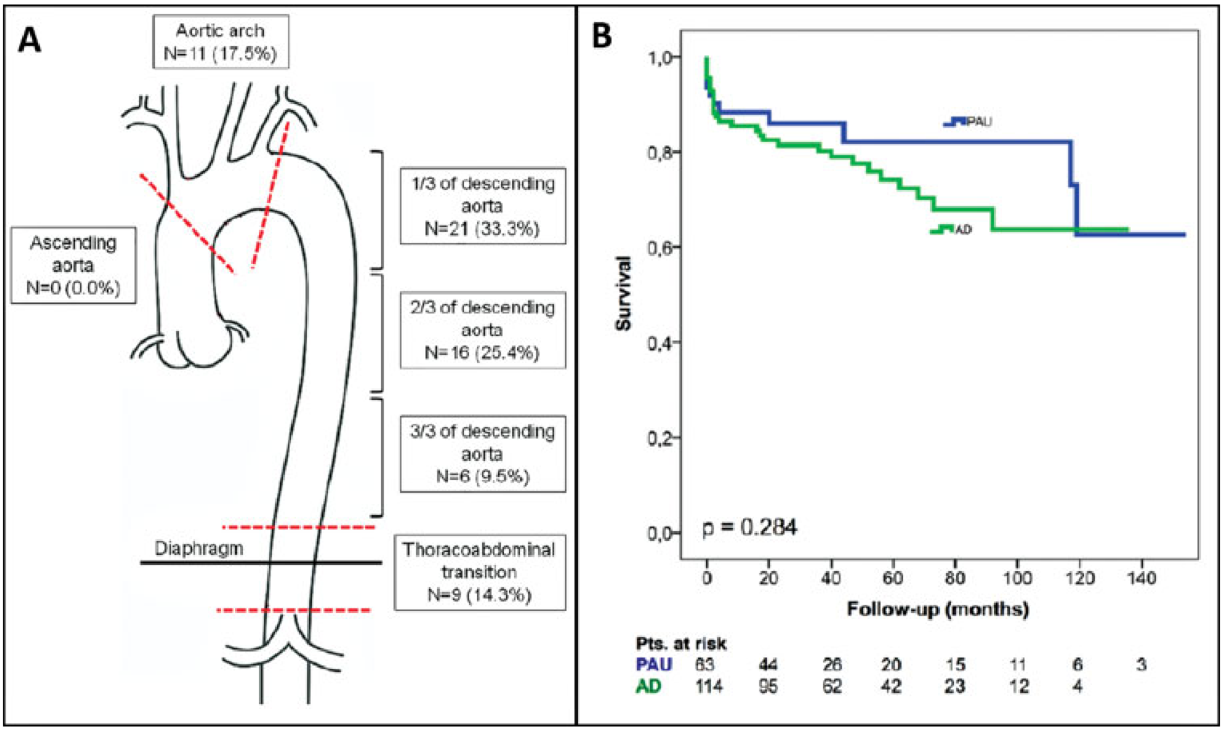

Supplementary Fig. S5 Distribution pattern and all-cause mortality estimates for patients with penetrating aortic ulcers (PAU) from the 11-year experience of the Mainz–Essen Centre. (**A**) Distribution pattern of PAUs. (**B**) All-cause mortality estimates for patients undergoing thoracic endovascular aortic repair for PAUs compared with patients with classic false lumen aortic dissection. The standard error is 8.6% after 122 months for the PAU group and 5.8% after 99 months for the AD group. Image courtesy: Jánosi et al.[5]

 © 2021. The Author(s).

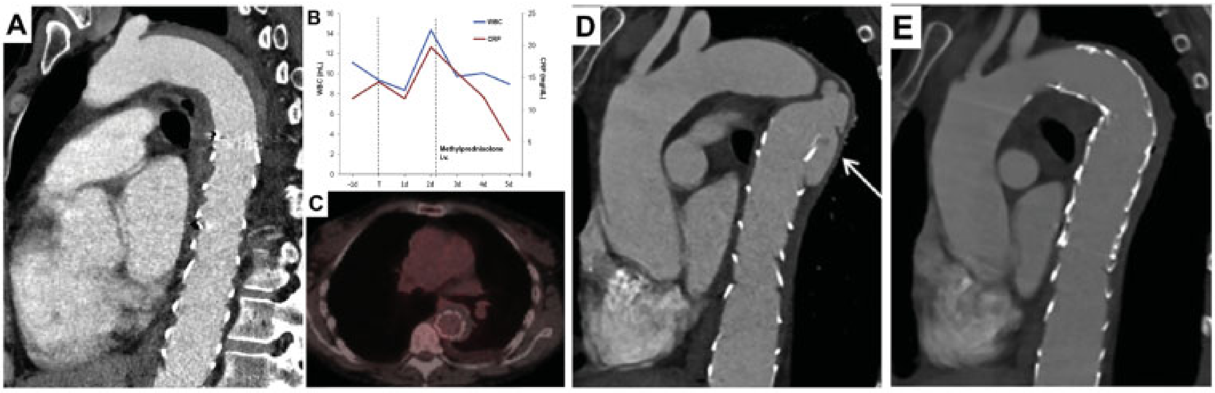

Supplementary Fig. S6 Postimplantation syndrome in a female patient after thoracic endovascular aortic repair. A 61-year-old female patient admitted with Type B acute aortic dissection underwent thoracic endovascular aortic repair (TEVAR). Postoperative computed tomography (CT) angiography showed successful closure of the intimal entry and thrombosis of the false lumen surrounding the stent-graft (**A**). 48 hours after TEVAR, the patient developed fever >38°C accompanied by back pain and tachycardia as well as a striking increase of serum levels of white blood cells (WBC) and C-reactive protein (CRP); blood culture results were negative and a diagnosis of post-implantation syndrome was made. The patient was treated with intravenous methylprednisolone with prompt regression of the symptoms and normalization of the inflammatory biomarkers (**B**). Positron emission tomography- CT imaging was performed pre-discharge and showed a modest ^{18}F-fluorodeoxyglucose uptake in the aortic wall surrounding the stent-graft (**C**). At 10-month follow-up, CT angiography showed a new intimal entry at the proximal edge of the stent-graft (**D,** arrow), which required implantation of another prosthesis (**E**). Image courtesy: Gorla et al.[61]

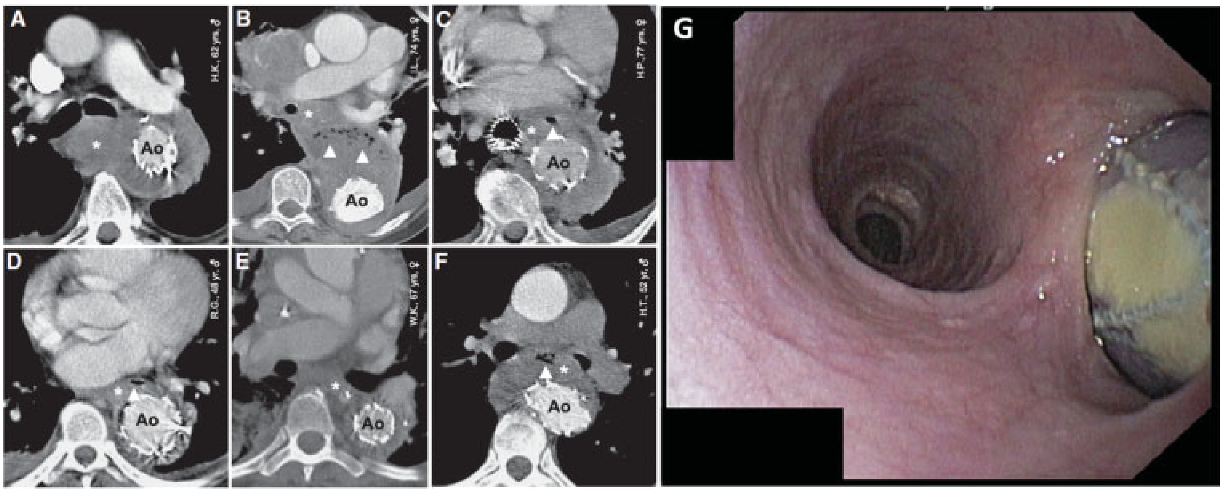

Supplementary Fig. S7 Aortoesophageal fistula characteristic appearance on computed tomography scans and on esophagogastroduodenoscopy. (**A to F**): Contrast-enhanced computed tomography (CT) scans of the six patients at presentation showing the characteristic appearance of an aortoesophageal fistula with evidence of new nonhomogeneous masses between the aorta and esophagus (asterisks). Note the air entrapment within the thrombosed aneurysm/dissection in (**B–F;** arrows). (**G**) Esophagogastroduodenoscopy demonstrating a large aortoesophageal fistula with an apparent view onto the intact aortic stent-graft. Ao, descending thoracic aorta. Image courtesy: Eggebrecht et al.[34]

 © 2021. The Author(s).

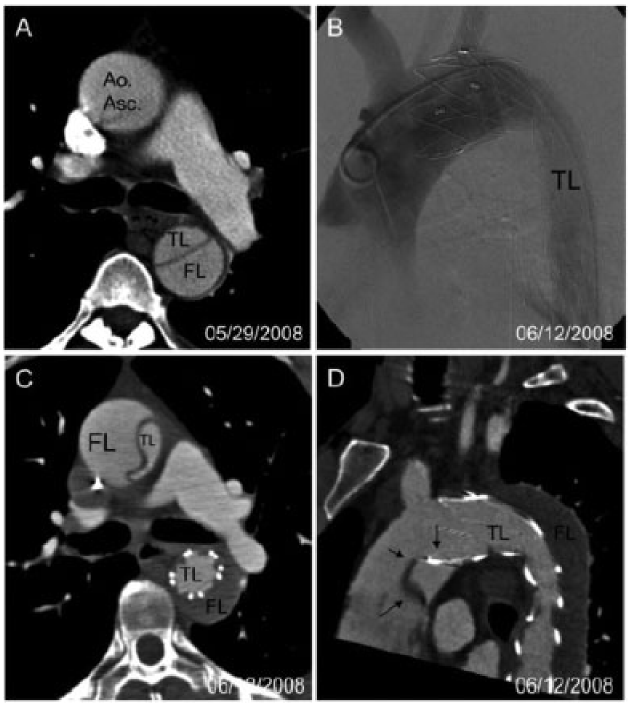

Supplementary Fig. S8 (**A**) Preoperative contrast-enhanced computed tomography (CT) scan showing Type B aortic dissection with true lumen (TL) and false lumen (FL) in the descending thoracic aorta. (**B**) Angiography after successful thoracic endovascular aortic repair (TEVAR) shows complete exclusion of the FL. (**C**) Contrast-enhanced CT 4 hours after TEVAR performed for sudden hemiplegia and aphasia shows retrograde ascending aortic dissection (rAAD) with perfused TL and FL. Note the stent graft and the completely thrombosed FL in the descending thoracic aorta. (**D**) Multiplanar CT reconstruction reveals stent-graft induced rAAD with ascending aortic injury related to the proximal bare spring of the endoprosthesis. Image courtesy: Eggebrecht et al.[40]

 © 2021. The Author(s).

Table S1 Review protocol and search strategy

**Review protocol and search strategy**
Review protocol:
A protocol for this review was prospectively developed, detailing specific objectives and criteria for study selection. For the study purpose, the Mainz–Essen experience on AAS was summarized by considering original articles from single-center or multicenter studies performed at West German Heart Centre, Essen, Germany or at the cardiovascular department of Johannes Gutenberg University, Mainz, Germany. Articles in German language, case reports, editorials, and reviews were excluded.
Search strategy:
An electronic search on MEDLINE was performed combining the search terms “Erbel R” or “Jakob H” (former chief cardiologist and chief heart surgeon, respectively, at West German Heart Centre in Essen) with “aortic disease,” “aortic dissection,” “intramural hematoma,” “penetrating aortic ulcer,” and “transection.” This search included studies published until December 2018. The search was supplemented by manually reviewing the reference list of the retrieved review articles. A flowchart of the study selection process is presented in ►**Fig. 1**.
Studies addressing surgery in patients with Type A AAD:
The search retrieved 21 studies (14 single-center and 7 multicenter); of them,10 were focused on FET,[6–15] 4 on E-vita,[16–19] 3 on dissectometer,[20–22] 1 on hybrid stent grafting,[23] 1 on angioscopy,[24] 1 on DeBakey’s classification,[25] and 1 on hybrid room[26] (►**Table 3**).
Studies addressing TEVAR outcome in patients with Type B AAS:
The search retrieved 17 studies (14 single center and 3 multicenter); of them, four were focused on outcome of TEVAR,[27–30] three on outcome in PAU,[5,31,32] three on aortoesophageal fistula,[33–35] one on descending thoracic aortic rupture,[36] one on acute renal failure,[37] one on aortic remodeling after TEVAR,[38] one on postoperative paraplegia,[39] one on retrograde dissection,[40] one on postoperative silent cerebral ischemia,[41] and one on thoracic aortic aneurysm expansion due to late distal stent-graft induced new entry[42] (►**Table 4**).

Abbreviations: AAD, acute aortic dissection; AAS, acute aortic syndrome; FET, frozen elephant trunk; PAU, penetrating aortic ulcer; TEVAR, thoracic endovascular aortic repair.

 © 2021. The Author(s).
